# Topographic Correlation of Histopathological Subtypes in Canine Mammary Tumors: Evidence of Non-Random Tumor Distribution

**DOI:** 10.3390/ani15172604

**Published:** 2025-09-05

**Authors:** Ana Hîruța, Alexandra Irimie, Vlad Ioan Bocăneț, Zoltán Miklós Gál, Alexandru Raul Pop, Claudiu Gal, Elvira Gagniuc, Cornel Cătoi

**Affiliations:** 1 Department of Veterinary Pathology, University of Agricultural Sciences and Veterinary Medicine, Calea Mănăștur 3-5, 400372 Cluj-Napoca, Romania; ana.hiruta@usamvcluj.ro (A.H.); cornel.catoi@usamvcluj.ro (C.C.); 2 Department of Anatomy, University of Agricultural Sciences and Veterinary Medicine, Calea Mănăștur 3-5, 400372 Cluj-Napoca, Romania; 3 Department of Manufacturing Engineering, Technical University of Cluj-Napoca, 400114 Cluj-Napoca, Romania; vlad.bocanet@tcm.utcluj.ro; 4 Department of Veterinary Obstetrics and Reproductive Pathology, University of Agricultural Sciences and Veterinary Medicine, Calea Mănăștur 3-5, 400372 Cluj-Napoca, Romania; zoltan.gal@usamvcluj.ro (Z.M.G.); alexandru.pop@usamvcluj.ro (A.R.P.); 5 Synevovet Laboratory, 021408 Bucharest, Romania; gal.claudiu@gmail.com (C.G.); elvira.gagniuc@fmvb.usamv.ro (E.G.); 6 Department of Pathology and Forensic Medicine, University of Agronomic Sciences and Veterinary Medicine of Bucharest, 011464 Bucharest, Romania

**Keywords:** mammary neoplasia, veterinary oncology, anatomical tumor patterns, histological grading

## Abstract

Canine mammary tumors (CMTs) are among the most frequently diagnosed neoplasms in female dogs, with complex etiologies involving age, breed, body size, and reproductive status. This retrospective study analyzed 250 cases comprising 361 mammary tumors to evaluate demographic patterns, histopathological subtypes, and their topographical distribution. The majority of tumors (88.64%) were malignant, with complex carcinoma being the most common subtype (24%), followed by intraductal papillary carcinoma (14.95%). The mean age at diagnosis was 9.36 years, with 60.8% of cases occurring in dogs older than 8 years. Mixed-breed dogs were most frequently affected, followed by Bichon-type breeds and German Shepherds. Small-sized dogs (<10 kg) accounted for 43.5% of cases. Regarding the reproductive status, 35.6% of dogs were unspayed, while the remainder were either intact or had an undetermined status, reflecting regional spaying practices. A statistically significant association was identified between histological subtype and mammary gland location, revealing non-random, region-specific tumor distribution. Complex carcinomas were frequently located in the left canial abdominal mammary segment (L.A2), right canial abdominal mammary segment (R.A2), and left inguinal mammary segment (L.ING); mixed and tubular carcinomas were more prevalent in R.A2 and R.ING; and tubulopapillary carcinomas appeared predominantly in L.ING. While no significant overall association was found between tumor grade and gland location, grade III carcinomas showed a possible predilection for the left inguinal gland (L.INGH), indicating a regional tendency that warrants further investigation. These findings underscore the importance of anatomical factors in the development of CMTs and support the integration of gland-specific tumor patterns into diagnostic assessment and surgical decision-making.

## Simple Summary

Mammary tumors are very common in female dogs and can significantly impact their health. This study reviewed 250 dogs with a total of 361 mammary tumors to understand the factors associated with their development and the locations where these tumors are most likely to occur. We examined age, breed, body size, and spaying status, as well as the types of tumors and their locations within the mammary chain. Most tumors were malignant, and the most frequent type was the complex carcinoma. Older dogs, especially those over 8 years, and small-sized breeds were more often affected. Mixed-breed dogs, Bichon-type breeds, and German Shepherds were the most commonly affected. We also found that some tumor types tend to develop in specific mammary glands rather than at random. This information can help veterinarians make more informed decisions about diagnosis and surgical planning, ultimately improving treatment outcomes for affected dogs.

## 1. Introduction

Cancer is a major health concern in dogs and continues to be one of the leading causes of mortality. Mammary cancer is the most commonly diagnosed malignancy in both women and female dogs [1,2]. Due to the clinical relevance of breast cancer, mammary gland tumors have been widely studied. As ethical limitations restrict human experimentation, animals with spontaneous mammary tumors serve as important models for developing in vitro systems and advancing cancer research [3].

Identifying reliable prognostic criteria for canine mammary tumors requires an integrated analysis of epidemiological, clinicopathological, and histopathological characteristics. Prognostic markers have been investigated in relation to several key variables, including tumor laterality (left vs. right mammary chain) [4], age, reproductive status (intact or spayed), tumor size [5], single or multiple tumors [6], histological type [7], benign versus malignant nature [8], histological grade and its prevalence in different mammary glands [9]. Such multifactorial analyses are essential for improving prognostication, guiding therapeutic decision-making, and advancing personalized treatment strategies in veterinary oncology.

This study aims to investigate gland-specific tumor distribution patterns that may help veterinarians identify which mammary glands are more prone to developing specific tumor types, including distinctions between benign and malignant forms and their metastatic potential. Although such patterns may support clinical decision-making, studies have reported inconsistencies between histopathological findings and the actual biological behavior or prognosis of certain tumors [10], with some canine mammary gland tumors (MGTs) potentially being misdiagnosed as benign on histological examination [11]. These discrepancies underscore the need for a more nuanced understanding of tumor localization and behavior, which can improve diagnostic accuracy, guide surgical decision-making, and enhance prognostic assessments.

## 2. Materials and Methods

### 2.1. Study Population

This study included a group of 250 female dogs diagnosed with spontaneous mammary gland tumors with no evidence of pulmonary metastasis. The dogs varied in age, breed, and reproductive status (intact or neutered) and had not received prior treatment for their mammary tumors.

### 2.2. Clinical Evaluation and Staging

Each participant underwent a thorough clinical evaluation and preoperative pulmonary radiographic assessment before undergoing either unilateral or bilateral mastectomy, along with the removal of the draining lymph nodes. The initial staging was determined during the clinical examination using the TNM grading system [12]. All mammary glands were examined macroscopically following the surgical treatment to assess localization. The clinical component of this study was carried out by veterinary clinicians as a therapeutic intervention. The study utilized tissue samples submitted for routine diagnostic purposes to the Pathology Department of the University of Agricultural Sciences and Veterinary Medicine Cluj-Napoca and SynevoVet Laboratories. 

Due to the diversity of breeds and the variability within the mixed-breed group, patients were categorized by body size—small (0–10 kg), medium (10–25 kg), large (25–45 kg), or giant (over 45 kg)—for more consistent analysis [13].

### 2.3. Tissue Collection and Histopathology

Tissue samples from each nodule were fixed in 10% neutral-buffered formalin, routinely processed, and embedded in paraffin wax for histopathological examination. The sections were then stained with hematoxylin and eosin (HE). The slides were then examined and diagnosed according to the Zappulli classification and grading system [14].

The tissue sections were examined using an Olympus BX-51 light microscope; the histopathological evaluation focused on determining the tumor’s histological type and grade and identifying neoplastic emboli within the blood and lymphatic vessels, both within the tumor and surrounding tissue.

### 2.4. Statistical Analysis

SPSS v26 software was used for the statistical analysis, and the dataset was prepared in Excel. A chi-square test of independence and Fisher’s exact test were performed to evaluate the statistical association between mammary gland tumor types and anatomical segments. To test whether the distribution of grades was associated with specific mammary regions, Pearson’s chi-square test of independence was conducted. The significance of the associations was interpreted based on *p*-values, with *p* < 0.01 considered highly significant, 0.01 ≤ *p* < 0.05 indicating a significant association, 0.05 ≤ *p* < 0.10 suggesting a weak association, and *p* ≥ 0.10 considered not significant. Adjusted residuals were calculated to identify which anatomical segments were more susceptible to specific tumor types. Residuals greater than two were considered statistically relevant, indicating cells with observed counts significantly different from expectations under the null hypothesis of independence. The strength of association was further assessed using Cramér’s V, with values interpreted as follows: 0.00–0.10 indicating a weak association, 0.10–0.30 indicating a moderate association, 0.30–0.50 indicating a strong association, and values > 0.50 representing a strong association.

## 3. Results

### 3.1. Epidemiology

This research included 250 female dogs and 361 mammary gland neoplasms. The mean age of the subjects was 9.36 years, with 60.8% being older than 8 years. Regarding reproductive status, 35.60% of the dogs were unspayed, 11.60% were spayed, and 52.80% were of unknown reproductive status. Out of the 250 female dogs included in the study, 23% (*n* = 57) were mixed-breed, 18% (*n* = 45) belonged to the Bichon variety (including Maltese, Bichon Frisé, and Bichon Havanais), and 9% *(n* = 23) were German Shepherds. A total of 193 dogs were purebred, while 57 were mixed-breed. The most common size category was that of small-breed dogs, accounting for 43.5% (108/250) of cases, followed by medium-sized breeds, 21.3% (53/250), large breeds, 12.5% (31/250), and giant breeds, 12.1% (30/250).

### 3.2. Macroscopical Analysis and Nodule Anatomical Distribution

Clinical examination revealed that 67.6% (*n* = 169) of cases presented with a single mammary nodule, while 30.4% (*n* = 76) exhibited multiple nodules, and 2% (*n* = 5) showed a diffuse distribution of masses. Mass distribution analysis showed that 84.8% of cases (*n* = 212) exhibited unilateral involvement, with 43.6% (*n* = 109) located on the left side and 41.2% (*n* = 103) on the right side. Bilateral distribution was observed in 15.2% (*n* = 38).

For statistical analysis of the tumor distribution in canine mammary glands, each anatomical segment was assessed individually using the following abbreviations: thoracic segment: L.T1 and R.T1 for the cranial thoracic glands (left and right, respectively); L.T2 and R.T2 for the caudal thoracic glands; abdominal segment—L.A1 and R.A1 for the cranial abdominal glands; L.A2 and R.A2 for the caudal abdominal glands; L.ING and R.ING for the left and right inguinal glands.

**Figure 1:**
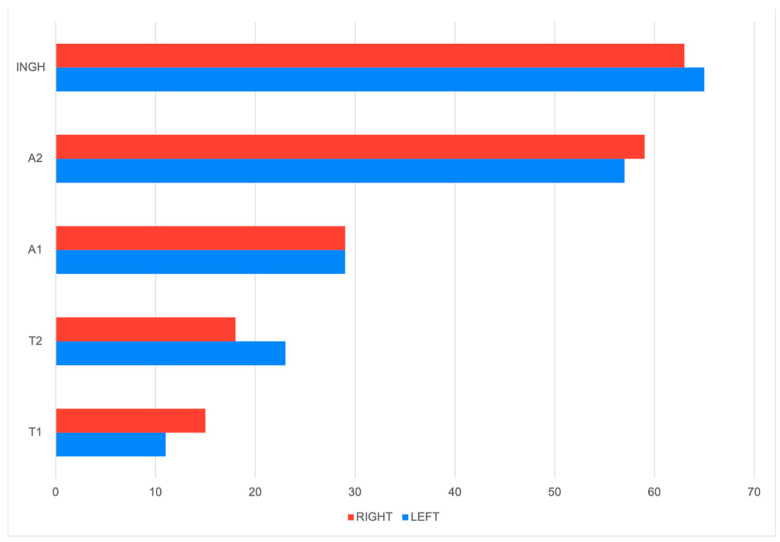
Comparison of tumor distribution across mammary glands by side (right vs. left).

Excluding cases with diffuse tumor distribution, nodules were nearly evenly distributed between the two mammary chains, with 181 tumors on the left side and 180 on the right. An increasing number of mammary gland tumors was observed from the cranial thoracic to the inguinal segments, with the lowest counts in T1 (left, right) and the highest in the inguinal glands (L.ING, R.ING), indicating a caudally progressive distribution pattern (Figure 1). While this difference was negligible at the chain level, segmental comparison revealed minor variations. Specifically, R.T1 exhibited a slightly higher number of tumors (*n* = 14) than L.T1 (*n* = 10), suggesting a marginally higher involvement of R.T1. Similarly, a slight difference was observed between L.T2 (*n* = 22) and R.T2 (*n* = 18). Despite these segment-level variations, no significant lateral asymmetry was identified when comparing the overall tumor burden between the left and right thoracic segments (L.T1, R.T1, L.T2, and R.T2 combined), indicating a relatively symmetrical distribution of tumors across the thoracic mammary regions.

The cranial abdominal segment showed no lateral difference, with an equal number of tumors on both sides (L.A1: *n* = 28, R.A1: *n* = 28). In contrast, the caudal abdominal segment exhibited a slight asymmetry, with a slightly lower count on the left (L.A2: *n* = 56) compared to the right (R.A2: *n* = 59). Similarly, minor differences were observed in the inguinal glands, where the left side (L.ING: *n* = 65) showed a marginally higher tumor frequency than the right (R.ING: *n* = 60).

**Figure 2:**
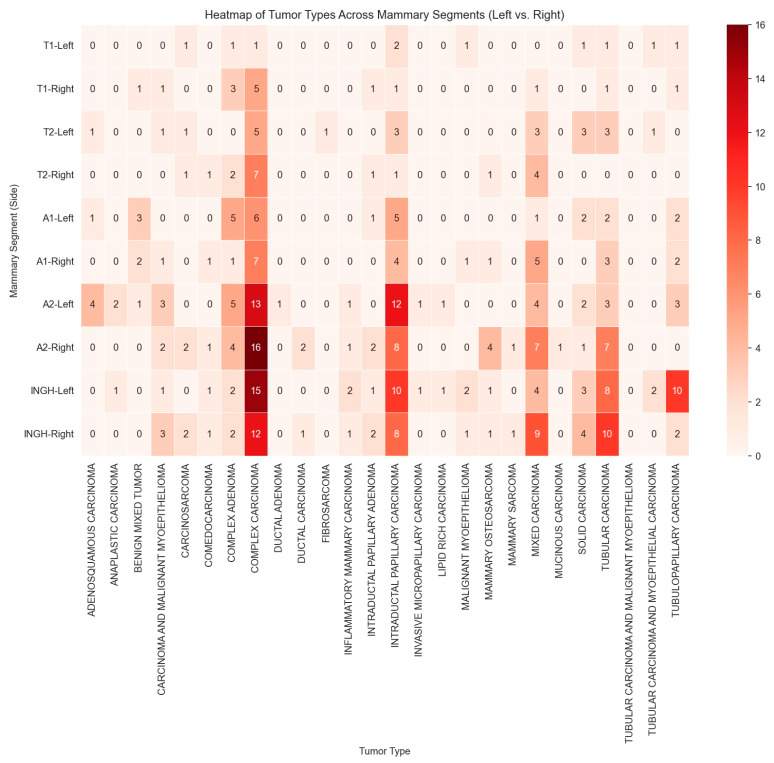
Heatmap showing the correlation between mammary tumor types and anatomical distribution (graphic generated using SPSS v26 software).

The analysis revealed a statistically significant association between tumor presence and mammary gland location (χ^2^(9) = 123.01, *p* < 0.001), indicating that the distribution of mammary gland tumors is not uniform along the mammary chain. This suggests that certain regions may be more predisposed to tumor development. The effect size (V = 0.222) indicates a small but meaningful association, implying that while gland location plays a role in tumor distribution, additional factors are likely to be involved in tumor pathogenesis (Figure 2).

Analysis of regional distribution revealed that L.ING (27.6%) and R.ING (26.0%) exhibited the highest proportions of tumor involvement, followed closely by L.A2 (24.8%) and R.A2 (24.8%). These values were significantly higher than expected under a uniform distribution model, with adjusted residuals exceeding +3.9, indicating a greater susceptibility of these regions to tumor development. Conversely, L.T1 (5.6%), R.T1 (8.0%), L.T2 (9.6%), and R.T2 (9.2%) showed the lowest frequencies of tumor occurrence. Adjusted residuals below (−3.0) confirmed that these regions were significantly less affected, suggesting reduced susceptibility or a potential protective effect in the cranial and caudal thoracic glands. Overall, these findings suggest a distinct anatomical pattern in tumor development, with the caudal abdominal and inguinal glands being significantly more vulnerable to tumor formation.

### 3.3. Histopathological Analysis and Tumor Subtypes’ Anatomical Distribution

Histological examination revealed that most tumors were malignant (88.64%, *n* = 320), while only 11.35% (*n* = 41) were classified as benign. No significant lateral difference was observed regarding the distribution of benign and malignant tumors within the mammary chains. On the left side, benign tumors accounted for 11.04% (20/181) of cases, while malignant tumors represented 88.95% (161/181). Similarly, on the right side, benign tumors constituted 11.66% (21/180), and malignant tumors were observed in 88.33% (159/180) of cases. These findings indicate a nearly symmetrical distribution of tumor types between the left and right mammary chains, with a consistently high prevalence of malignant lesions on both sides.

**Figure 3:**
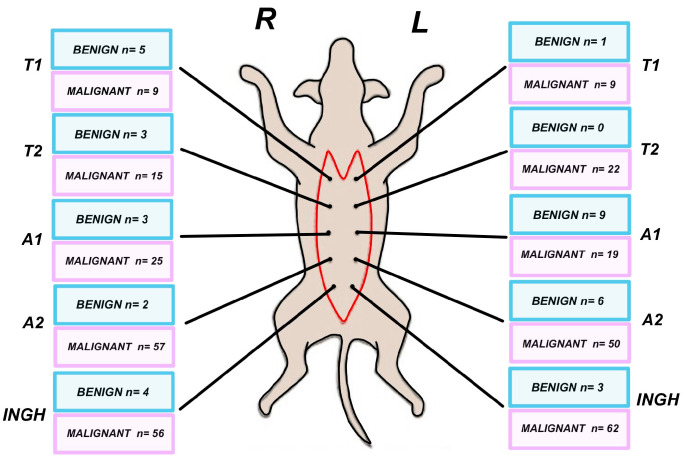
Benign and malignant distribution of the cases.

In the T1 segment, malignant tumors dominate at 2.49% on both the left and right sides, while benign cases are considerably lower, at 0.27% on the left and 1.38% on the right. T2 shows no benign cases on the left and only 0.83% on the right, whereas malignant cases constitute 6.09% on the left and 4.15% on the right. For A1, benign cases make up 2.49% (L.A1) and 0.83% (R.A1), with malignant cases at 5.26% (L.A1) and 6.92% (R.A1). The trend continues with the A2 segment, where malignant cases account for 13.85% of L.A2 and 15.78% of R.A2, compared to benign cases at only 1.66% and 0.55%, respectively. Lastly, the inguinal region has the highest malignant occurrence overall—14.40% on L.ING and 15.51% on R.ING—while benign cases are relatively rare, with prevalences of 0.83% (L.ING) and 1.10% (R.ING). These figures highlight a strong association between the A2 and ING segments and malignancy, suggesting their potential significance in treatment planning (Figure 3).

A total of 25 distinct histological tumor types were identified. Of the 250 cases evaluated, 80.4% (*n* = 201) presented with a single tumor type, while 19.6% (*n* = 49) exhibited multiple tumor types within the same patient. Notably, 16% of cases (*n* = 40) showed the presence of tumor emboli, indicating vascular invasion and a greater potential for metastatic spread.

Complex carcinoma was the most frequently identified tumor type, accounting for 24% of cases (*n* = 87). This was followed by intraductal papillary carcinoma, observed in 14.95% of cases (*n* = 54). Tubular carcinoma and mixed carcinoma were each diagnosed in 10.52% of cases (*n* = 38). Complex adenoma was identified in 6.92% (*n* = 25) of cases, while tubulopapillary carcinoma was present in 5.81% (*n* = 21). As summarized in Table 1, the remaining tumor types were observed in less than 5% of cases.

The analysis revealed a statistically significant association between the mammary gland region and tumor type occurrence, indicating that tumor type distribution is not random and that certain regions are more frequently affected. Regional analysis of complex carcinoma cases showed the highest proportions of complex carcinoma occurrence in R.A2, 24.1%, L.ING, 22.8%, and L.A2, 20.3% (χ^2^(9) = 24.64, *p* = 0.003, V = 0.177). These frequencies were higher than expected (adjusted residuals of 2.2 for L.ING, 2.6 for R.A2, and 1.6 for L.A2).

**Figure 4:**
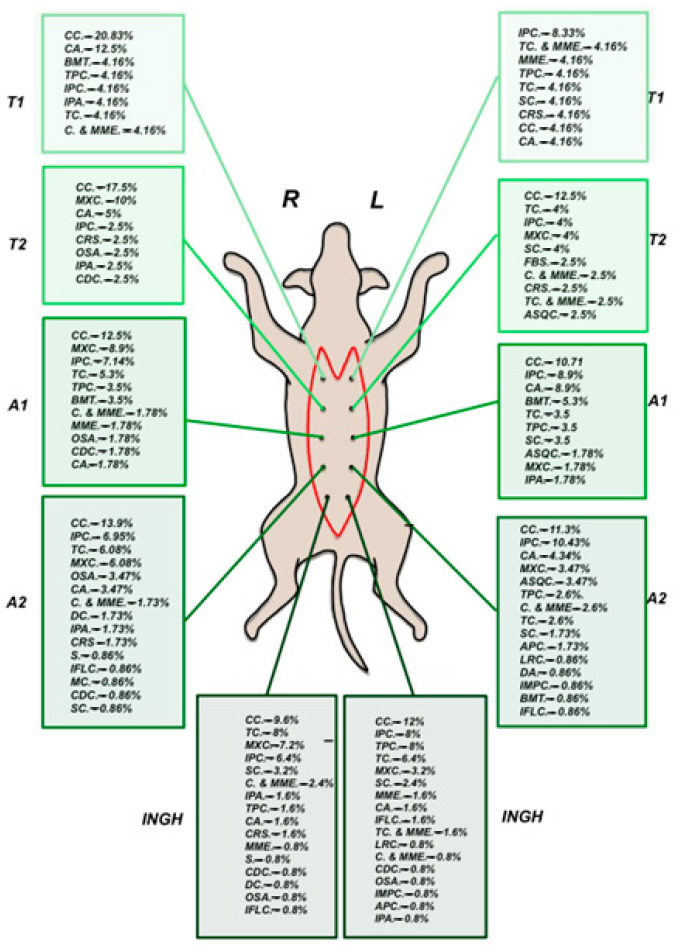
Incidence of mammary tumor types across anatomical gland segments. Legend: APC = anaplastic carcinoma; ASQC = adenosquamous carcinoma; BMT = benign mixed tumor; C = mammary carcinoma; CA = complex adenoma; CC = complex carcinoma; CDC = comedocarcinoma; DA = ductal adenoma; DC = ductal carcinoma; FBS = fibrosarcoma; IFLC = inflammatory carcinoma; IMPC = invasive micropapillary carcinoma; IPA = intraductal papillary adenoma; IPC = intraductal papillary carcinoma; LRC = lipid-rich carcinoma; MXC = mixed carcinoma; MC = mucinous carcinoma; MME = malignant myoepithelioma; OSA = mammary osteosarcoma; S = mammary sarcoma; SC = solid carcinoma; TC = tubular carcinoma; TPC = tubulopapillary carcinoma.

In cases of intraductal papillary carcinoma, the analysis revealed a statistically significant association between mammary gland region and tumor occurrence (χ^2^(9) = 24.67, *p* = 0.003, Cramér’s V = 0.229), indicating a small-to-moderate strength of association. The L.A2 and L.ING glands exhibited a higher-than-expected prevalence of intraductal papillary carcinoma. Specifically, L.A2 showed the highest occurrence (27.7%), significantly exceeding expected values (adjusted residual = 3.0), while L.ING also demonstrated an elevated prevalence (23.4%, adjusted residual of 2.1) (Figure 4).

Cases presenting with the mixed carcinoma subtype showed a borderline statistically significant association between mammary gland region and tumor occurrence (χ^2^(9) = 16.81, *p* = 0.052, Fisher’s exact test *p* = 0.053, Cramér’s V = 0.219), suggesting a small-to-moderate association. The analysis revealed that the R.ING (28.6%) and R.A2 (22.9%) mammary glands exhibited a higher-than-expected occurrence of mixed carcinoma (adjusted residual of 2.7).

The results revealed a statistically significant association between the mammary gland region and the occurrence of tubular carcinoma (χ^2^(9) = 29.89, Fisher’s exact test *p* < 0.001, Cramér’s V = 0.288), indicating a moderate strength of association. R.ING and R.A2 glands had significantly higher-than-expected occurrences of tubular carcinoma. R.ING showed the highest prevalence (27.8%), markedly exceeding expected values (adjusted residual = 3.5). Regarding tubulopapillary carcinoma cases, the analysis showed a significant association between the mammary region and tumor occurrence (χ^2^(9) = 43.25, *p* < 0.001), with a notable concentration in L.ING, where prevalence reached 62.5% (adjusted residual = 6.2), well above expected levels.

#### 3.3.1. Tumor Grading

Among all carcinomas, grade I tumors were the most common (44.65%), followed by grade III (27.86%) and grade II (27.48%) tumors (Table 2).

Overall, there was no clear pattern indicating a relationship between tumor grade and specific mammary gland regions (χ^2^(18) = 22.37, Pearson’s chi-square *p* = 0.216). However, grade III tumors appeared to occur more frequently in L.INGH (residuals exceeding ±2), suggesting a potential regional tendency.

#### 3.3.2. TNM Score

All cases were initially clinically staged using the TNM system, with the final stage determined after histopathological evaluation of the lymph nodes. The T score was assigned in cases with multiple nodules based on the largest tumor (Table 3).

## 4. Discussion

The present study revealed a statistical association concerning the histopathological subtypes of canine mammary gland tumors and their anatomical site of occurrence. To the best of the authors’ knowledge, this is the first study to investigate potential anatomical patterns by examining whether specific histopathological subtypes of canine mammary tumors are more likely to develop in particular mammary glands. This gland-targeted approach provides a novel contribution that goes beyond general histopathological classification and may assist veterinarians in better understanding tumor distribution and risk. The authors acknowledge the limitations of this study, which should be regarded as a preliminary step toward a broader investigation that includes a larger cohort of tumors.

Our results support existing evidence that malignant mammary tumors in female dogs are most commonly diagnosed in middle-aged to older individuals. The median age of occurrence is typically between 8 and 10 years [15], with the highest frequency seen in dogs aged 9–12 years, followed by those aged 5–8 years [16]. In the present study, the mean age was 9.36 years, with 60.8% of cases occurring in dogs older than 8 years, reinforcing the age-related pattern observed in earlier research.

Certain breeds—such as Poodles, English Springer Spaniels, Brittany Spaniels, Cocker Spaniels, German Shepherds, Maltese, Yorkshire Terriers, and Dachshunds—have been reported to be at higher risk of developing CMTs [16]. Purebred dogs have also been significantly overrepresented among CMT cases [17]. A study from Sweden reported a higher incidence in German Shepherds, English Springer Spaniels, and Golden Retrievers [18], while Salas et al. also noted increased rates in mixed-breed dogs, Beagles, and Poodles. Similarly, a large-scale cohort study by Rafalko et al. identified Golden Retrievers, Labrador Retrievers, and Boxers as the breeds with the highest predisposition for mammary tumors [19]. In our study, mixed-breed dogs represented the most significant proportion of cases, followed by Bichon-type dogs and German Shepherds. Similarly, Ariyarathna et al. (2018) [20] reported that 39 of 74 dogs (approximately 50%) diagnosed with mammary tumors were mixed breeds. Among the purebred dogs in that study, German Shepherds were the most common (*n* = 21; 28.4%), followed by Dachshunds (*n* = 2; 2.7%) [20]. However, breed distribution reflects the general dog population and breed prevalence in a given region rather than true breed predisposition, though genetic factors are likely to contribute to an increased risk of CMTs.

The relationship between body size and the occurrence of canine mammary tumors (CMTs) has been explored in several studies with varying results. A study from China reported that 56.86% of single tumors occurred in large-breed dogs, while multiple tumors were most prevalent in medium-sized dogs (48.43%, 62/128) [21]. More consistent with our findings, other studies have indicated a higher incidence of CMTs in small dogs, typically defined as dogs weighing less than 20–23 kg [7,8,18]. In our study, we defined small dogs as those weighing less than 10 kg and found that 43.5% of CMT cases occurred in this category, suggesting a potential predisposition. However, this result may also reflect the general dog population in the study area, where small breeds are more commonly represented.

Reproductive status is a well-established risk factor for the development of CMTs. In a study by Pastor et al., only 14.71% of affected dogs were spayed at the time of diagnosis. Similarly, Zheng et al. reported that approximately 75.2% of CMT cases occurred in unspayed females. In our study, 35.6% of the dogs were unspayed, while 64.4% were either intact or had an undetermined reproductive status. In Romania, most geriatric female dogs remain intact, as early spaying has only recently become a common practice. These differences likely reflect regional variations in population demographics and veterinary practices, underscoring the protective role of early spaying [22]. The incomplete information on reproductive status represents a limitation of our study, as it reduces the precision with which the impact of spaying on tumor development can be assessed.

The present study shows that the majority of canine tumors were malignant (88.64%), a rate comparable to that reported in Sri Lanka (88%) [20] and Brazil (86%) [23]. Malignant mammary gland tumors (MGTs) may be overrepresented in these countries due to dogs being more frequently exposed to carcinogens that may not be prevalent in countries like the USA, Canada, Japan, and Mexico [20]. In Europe, a study performed in Spain showed that 89.2% of mammary tumors were malignant [24], while studies showed the opposite results: in Portugal, 53.1% were classified as benign tumors [25], as well as one performed in the UK [26]. In Europe, the mixed results between countries like Spain, Romania, Portugal, and the UK may reflect differences in healthcare practices, genetic backgrounds, environmental factors, and public awareness. Countries with more advanced veterinary healthcare systems and proactive spaying policies tend to report higher rates of benign tumors, while others with different management practices may see more malignant cases. These discrepancies highlight the importance of genetic, environmental, and healthcare-related factors in developing and detecting mammary tumors in dogs worldwide.

A study conducted in Sri Lanka shows that among all malignant tumors, simple carcinoma comprised 17.6% and mixed carcinoma comprised 13.5% [20]. In Mexico, where most tumors were benign, within the malignant group, adenocarcinoma was reported as the most common lesion (52.3%), followed by mixed tumors (44.7%) [16]. In another study, the two most frequent types of malignant tumors diagnosed were complex carcinoma (42.30%) and tubulopapillary carcinoma (29.74%) [16]. Mixed carcinomas were the most frequently observed malignant tumors in one study (27.4%), followed by complex carcinomas (19%) and tubulopapillary carcinomas (17.8%) [27]. In the present study, complex carcinoma was the most frequently identified tumor type (24%) of cases, followed by intraductal papillary carcinoma (14.95%). A limitation of this study is the low number of cases recorded for certain histological subtypes, which limits the statistical power for drawing conclusions about these categories. However, their inclusion was essential, as the aim of this research was to investigate gland-specific tumor distribution patterns, including rare types. The low case numbers reflect the actual rarity of these histological subtypes, which are also classified as special types by Zappulli et al., and provide valuable insight into the full histopathological spectrum encountered in clinical practice.

Differences in histopathological classification systems, misdiagnoses, and variability between institutions and pathologists can significantly affect clinical data interpretation that depends on histological findings as the standard reference [11]. One study emphasized that histological diagnosis remains essential and cannot be replaced by grading systems, which are only meant to complement it [7]. Despite the limited number of cases for specific tumor types, histological diagnosis was shown to have significant prognostic value in this prospective study through univariate analyses [7]. Building on this, our aim to investigate gland-specific tumor distribution patterns could help veterinarians identify which mammary glands are more prone to developing particular tumor types, thereby enhancing treatment planning and prognostic accuracy.

The current study found no significant differences in the topographical distribution of benign and malignant tumors between the left and right mammary chains. While the proportion of benign tumors remains relatively stable from cranial to caudal glands, the incidence of malignant tumors increases progressively along the mammary chain in a cranial-to-caudal direction, similar to recent studies [9].

A study reported that 58.8% of tumors were located on the left mammary chain and 41.2% on the right, though their findings were based on a limited number of cases [28]. Similarly, in another study, researchers observed a slight predominance on the left side, with 57.14% (*n* = 32) of malignant tumors occurring on the left and 42.86% (*n* = 24) on the right [4]. Regarding anatomical distribution, their study showed the highest tumor frequency in the fifth mammary gland (42.86%), followed by the fourth (26.78%), third (17.86%), second (8.93%), and first (3.57%). Another study reported a similar trend, with tumors more frequently affecting the caudal glands: 5.76% in the first, 10.31% in the second, 27.38% in the third, 30.56% in the fourth, and 25.99% in the fifth mammary gland [21]. In contrast, our study did not find a significant difference in tumor occurrence between the right and left mammary chains. Our findings are consistent with the caudal predominance of tumor distribution; however, to the author’s knowledge, this is the first study to examine tumor distribution in direct correlation with histopathological subtypes.

This study identified a statistically significant association between the anatomical location of the mammary gland and the histological type of the tumor, suggesting that the tumor distribution along the mammary chain may not be random. Certain tumor types were more frequently associated with specific glandular regions. Complex carcinomas showed a tendency to occur in the L.A2, R.A2, and L.ING glands. Intraductal papillary carcinomas were predominantly observed on the left side, particularly in L.A2 and L.ING. Mixed carcinomas demonstrated a right-sided pattern, being more commonly found in R.A2 and R.ING. Tubular carcinomas were primarily located in the right abdominal and right inguinal glands (R.A2 and R.ING), while tubulopapillary carcinomas appeared predominantly in the left inguinal gland (L.ING). These findings are significant, as they suggest a potential relationship between tumor location, histological subtype, and biological behavior. Importantly, existing literature indicates that low-grade, well-differentiated carcinomas, including complex, simple tubular, and simple tubulopapillary types, are associated with a better prognosis [29]. More aggressive tumor types, including carcinosarcomas and anaplastic carcinomas, tend to have poorer outcomes and may not exhibit such a distinct regional distribution pattern [29]. The observation that certain tumor subtypes tend to localize preferentially in specific glands could offer valuable insights into their behavior and prognosis. Recognizing these patterns may enhance diagnostic accuracy and surgical planning, ultimately contributing to improved prognostic assessments.

Several factors may influence the observed variation in tumor distribution across different mammary gland regions. Anatomically, certain glands may have a higher density of mammary tissue or more extensive ductal systems [28], making them more susceptible to specific tumor types. Exposure to exogenous or pharmacological concentrations of sex hormones, particularly progestins and estrogens, has been shown to increase the risk of mammary tumor development in dogs [30]. In addition, hormonal influences may contribute to the anatomical distribution of tumors, as regional differences in tissue density could result in variable responsiveness to hormonal fluctuations, thereby influencing tumor growth patterns.

Additionally, local microenvironmental factors, such as blood supply, tissue composition, or carcinogen exposure [31], might predispose some glands to specific tumor types. Genetic or breed predispositions could further contribute to regional differences, as certain genetic traits may influence tumor susceptibility in specific mammary glands. Understanding these factors and tumor behavior could support more targeted clinical approaches, such as imaging and especially biopsy, which is particularly valuable for early detection or in cases with small or multifocal tumors [32]. It also informs surgical decision-making, including the extent of mastectomy (regional versus radical) [33], the prophylactic removal of adjacent glands, and the need for lymph node dissection based on lymphatic drainage pathways [34]. By examining potential anatomical patterns across histological subtypes, this work provides a basis for exploring why certain regions are more prone to particular tumor types. Further investigations integrating molecular, genetic, and environmental perspectives will be essential to deepen the understanding of these patterns and to refine diagnostic and therapeutic strategies in veterinary oncology. Given the observed regional predilections for specific tumor histopathological subtypes identified in this study, selecting an appropriate surgical strategy is critical for optimizing clinical outcomes [35]. Previous studies have reported that mammary tumor types differ in their invasiveness, metastatic potential, and risk of recurrence [29]. These characteristics underscore the importance of tailoring the surgical approach to the biological behavior of the tumor. Our findings regarding anatomical distribution patterns may therefore provide additional guidance when determining the extent of surgical intervention, whether it takes the form of a nodulectomy, regional mastectomy, or complete unilateral mastectomy. Although a double mastectomy is generally recommended in cases of mammary gland tumors due to its comprehensive nature, it remains a radical surgical approach associated with an extended recovery period [30]. Given that the majority of affected patients are over 8 years old and frequently present with increased anesthetic risk and prolonged wound healing [36], our findings support the consideration of a partial mastectomy in selected, high-risk cases. Traditionally, radical mastectomy has been considered the most reliable option to minimize the risk of local recurrence [33]. Nevertheless, recent studies have highlighted that the key determinant of survival is the completeness of excision, rather than the extent of tissue removed [33]. In this context, our findings support the consideration of partial or regional mastectomy in carefully selected, high-risk cases, where achieving macroscopically complete resection is feasible and the anatomical distribution of tumors justifies a more conservative approach. This perspective introduces an alternative to the traditional radical strategy, and it may warrant further prospective evaluation.

The present study is constrained by sparse tumor counts per mammary gland, notably in thoracic segments, and the limited representation of certain histopathologic subtypes, which may affect statistical power and the reliability of subtype-level inferences. Replication in a larger cohort is likely to enhance the clarity of the observed patterns, rendering them more discernible. An expanded analysis would also yield a more robust characterization of tumor-grade distributions, enabling a more precise assessment of grade-related trends across glandular sites. A further limitation is the incomplete reproductive data, as information on age at castration and, in some cases, reproductive status was not systematically provided by the submitting clinicians, thereby limiting the capacity to fully assess their potential influence on tumor development. To advance understanding in this area, multi-institutional, prospective studies with larger and more diverse cohorts are needed to confirm gland-specific patterns, refine subtype- and site-related risk estimates, and incorporate key reproductive variables. Future work should also explore potential interactions between histopathologic subtype, anatomical site, and grade, and consider environmental, reproductive, and genetic risk factors to inform risk stratification and targeted management.

## 5. Conclusions

This study identifies statistically significant gland-specific patterns in the histopathologic subtypes of canine mammary tumors, thereby adding a novel dimension to the characterization of tumor distribution beyond conventional histologic classification. The observed patterning provides a basis for refined risk stratification and for informing targeted surveillance and therapeutic strategies in canine mammary neoplasia.

## Figures and Tables

**Table 1:** Histological classification and tumor counts in left versus right mammary chains.

Histological Type	Number of Tumors on the Left Mammary Chain	Number of Tumors on the Right Mammary Chain	Total
Complex Carcinoma	40	47	87
Intraductal Papillary Carcinoma	32	22	54
Tubular Carcinoma	17	21	38
Mixed Carcinoma	12	26	38
Complex Adenoma	13	12	25
Tubulopapillary Carcinoma	16	5	21
Solid Carcinoma	11	5	16
Carcinoma And Malignant Myoepithelioma	5	7	12
Mammary Osteosarcoma	1	7	8
Intraductal Papillary Adenoma	2	6	8
Benign Mixed Tumor	4	3	7
Carcinosarcoma	2	5	7
Adenosquamous Carcinoma	6	0	6
Malignant Myoepithelioma	3	2	5
Inflammatory Mammary Carcinoma	3	2	5
Comedocarcinoma	1	4	5
Tubular Carcinoma And Myoepithelial Carcinoma	4	0	4
Ductal Carcinoma	0	3	3
Anaplastic Carcinoma	3	0	3
Mammary Sarcoma	0	2	2
Invasive Micropapillary Carcinoma	2	0	2
Lipid-Rich Carcinoma	2	0	2
Fibrosarcoma	1	0	1
Mucinous Carcinoma	0	1	1
Ductal Adenoma	1	0	1

**Table 2:** Topographical distribution of tumor grades along the mammary chains.

	T1			T2			A1			A2			INGH		
GRADE (*n*)	I	II	III	I	II	III	I	II	III	I	II	III	I	II	III
LEFT	2	2	2	7	8	3	8	5	5	14	16	13	22	8	20
RIGHT	6	3	1	8	2	3	10	5	4	21	12	7	19	11	15

**Table 3:** TNM score and clinical staging of cases with mammary gland tumors.

TNM Score (*n*)	Clinical Stage (%)	Number of Patients	
T1N0M0	1	130 (52%)	
T2N0M0	2	40 (1.6%)	
T3N0M0	3	53 (21.2%)	
T1N1M0	4	5 (2%)	25 (10%)
T2N1M0		4 (1.6%)	
T3N1M0		16 (6.4%)	
T3N1M1	5	2 (0.8%)	

## Data Availability

The raw data supporting the conclusions of this article will be made available by the authors on request.

## Abbreviations

The following abbreviations are used in this manuscript:

MGT	Mammary Gland Tumor.
CMT	Canine Mammary Tumor.
TNM	Tumor Node Metastasis.
HE	Hematoxylin and Eosin.
L.T1	Left Cranial Thoracic Mammary Segment.
L.T2	Left Caudal Thoracic Mammary Segment.
L.A1	Left Cranial Abdominal Mammary Segment.
L.A2	Left Cranial Abdominal Mammary Segment.
L.ING	Left Inguinal Mammary Segment.
R.T1	Right Cranial Thoracic Mammary Segment.
R.T2	Right Caudal Thoracic Mammary Segment.
R.A1	Right Cranial Abdominal Mammary Segment.
R.A2	Right Caudal Abdominal Mammary Segment.
R.ING	Right Inguinal Mammary Segment.
APC	Anaplastic Carcinoma.
ASQC	Adenosquamous Carcinoma.
BMT	Benign Mixed Tumor.
C	Mammary Carcinoma.
CA	Complex Adenoma.
CC	Complex Carcinoma.
CDC	Comedocarcinoma.
DA	Ductal Adenoma.
DC	Ductal Carcinoma.
FBS	Fibrosarcoma.
IFLC	Inflammatory Carcinoma.
IMPC	Invasive Micropapillary Carcinoma.
IPA	Intraductal Papillary Adenoma.
IPC	Intraductal Papillary Carcinoma.
LRC	Lipid-Rich Carcinoma.
MXC	Mixed Carcinoma.
MC	Mucinous Carcinoma.
MME	Malignant Myoepithelioma.
OSA	Mammary Osteosarcoma.
S	Mammary Sarcoma.
SC	Solid Carcinoma.
TC	Tubular Carcinoma.
TPC	Tubulopapillary Carcinoma.
